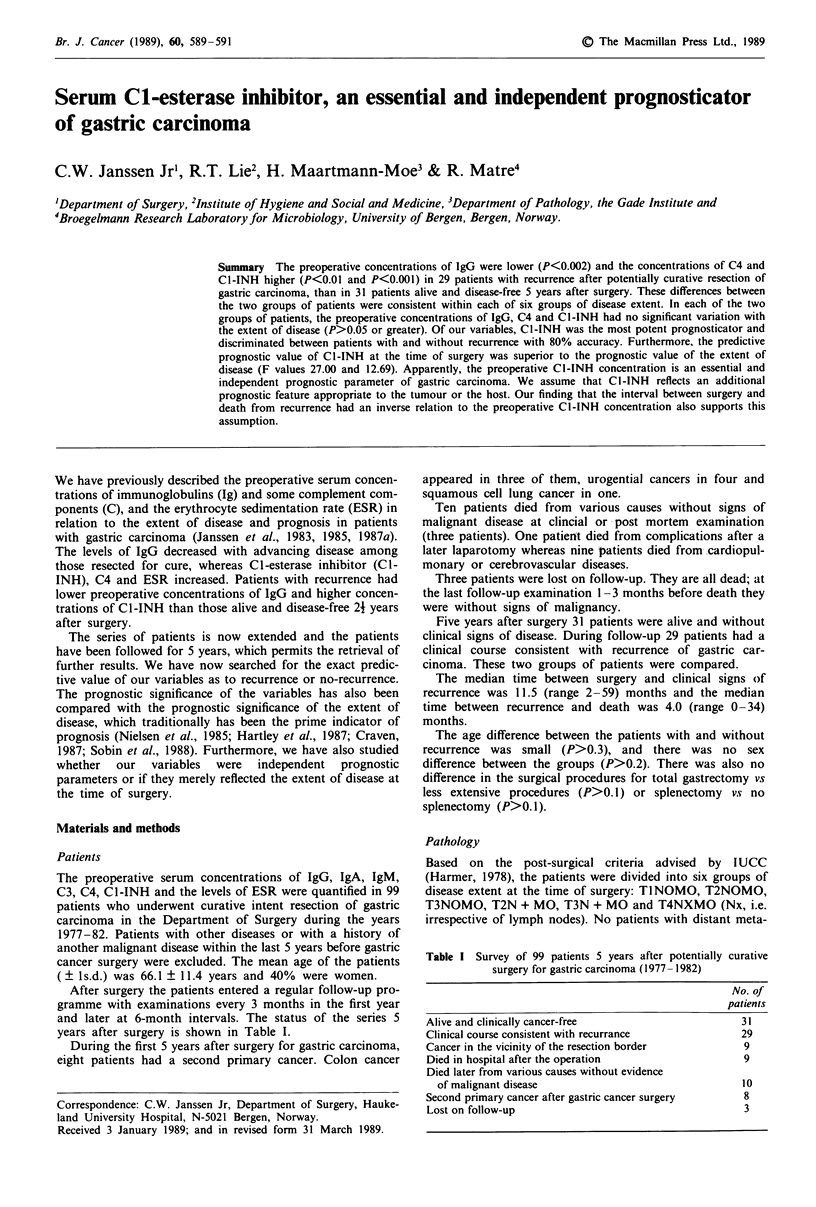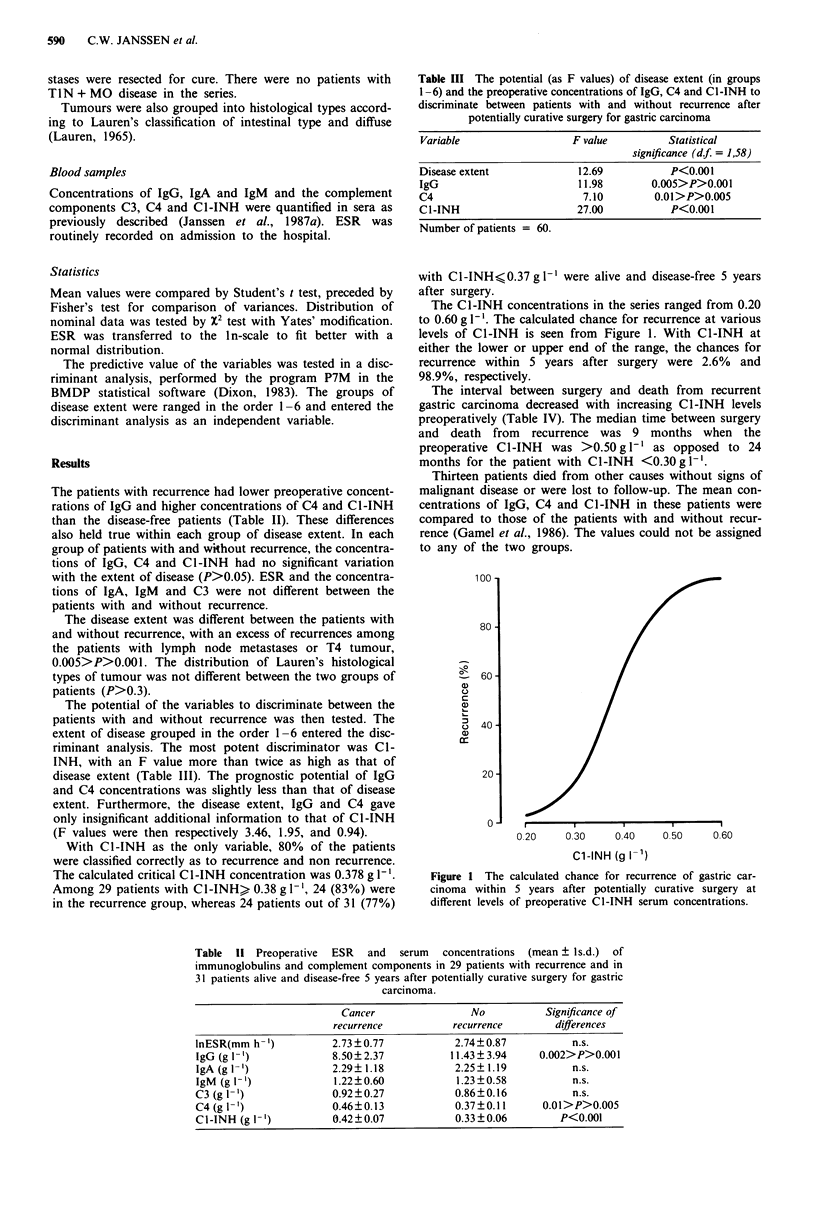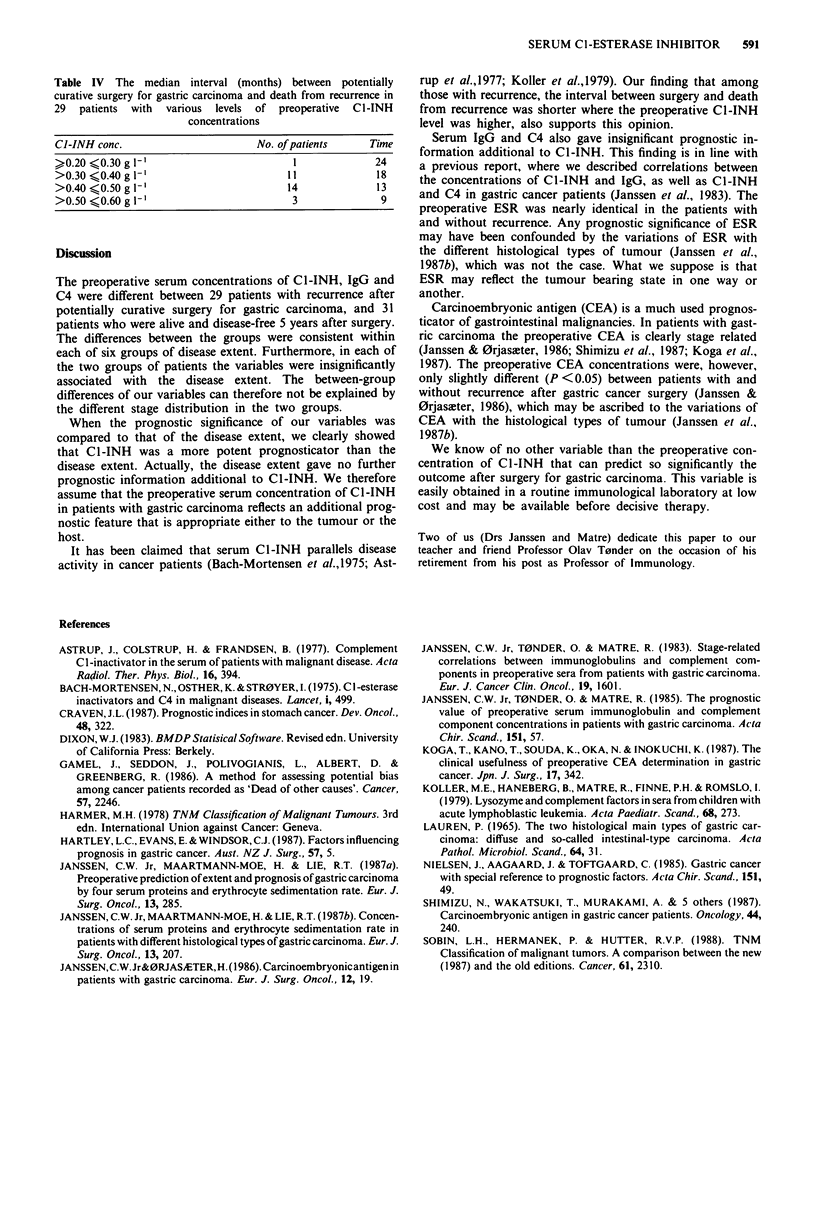# Serum C1-esterase inhibitor, an essential and independent prognosticator of gastric carcinoma.

**DOI:** 10.1038/bjc.1989.319

**Published:** 1989-10

**Authors:** C. W. Janssen, R. T. Lie, H. Maartmann-Moe, R. Matre

**Affiliations:** Department of Surgery, University of Bergen, Norway.

## Abstract

The preoperative concentrations of IgG were lower (P less than 0.002) and the concentrations of C4 and C1-INH higher (P less than 0.01 and P less than 0.001) in 29 patients with recurrence after potentially curative resection of gastric carcinoma, than in 31 patients alive and disease-free 5 years after surgery. These differences between the two groups of patients were consistent within each of six groups of disease extent. In each of the two groups of patients, the preoperative concentrations of IgG, C4 and C1-INH had no significant variation with the extent of disease (P greater than 0.05 or greater). Of our variables, C1-INH was the most potent prognosticator and discriminated between patients with and without recurrence with 80% accuracy. Furthermore, the predictive prognostic value of C1-INH at the time of surgery was superior to the prognostic value of the extent of disease (F values 27.00 and 12.69). Apparently, the preoperative C1-INH concentration is an essential and independent prognostic parameter of gastric carcinoma. We assume that C1-INH reflects an additional prognostic feature appropriate to the tumour or the host. Our finding that the interval between surgery and death from recurrence had an inverse relation to the preoperative C1-INH concentration also supports this assumption.


*Br. J. Cancer* (1989), **60**, 589–591 © The Macmillan Press Ltd., 1989

### Serum C1-esterase inhibitor, an essential and independent prognosticator of gastric carcinoma

C.W. Janssen Jr[1], R.T. Lie[2], H. Maartmann-Moe[3] & R. Matre[4]

[1]*Department of Surgery,* [2]*Institute of Hygiene and Social and Medicine,* [3]*Department of Pathology, the Gade Institute and* [4]*Broegelmann Research Laboratory for Microbiology, University of Bergen, Bergen, Norway.*

**Summary** The preoperative concentrations of IgG were lower ($P<0.002$) and the concentrations of C4 and C1-INH higher ($P<0.01$ and $P<0.001$) in 29 patients with recurrence after potentially curative resection of gastric carcinoma, than in 31 patients alive and disease-free 5 years after surgery. These differences between the two groups of patients were consistent within each of six groups of disease extent. In each of the two groups of patients, the preoperative concentrations of IgG, C4 and C1-INH had no significant variation with the extent of disease ($P>0.05$ or greater). Of our variables, C1-INH was the most potent prognosticator and discriminated between patients with and without recurrence with 80% accuracy. Furthermore, the predictive prognostic value of C1-INH at the time of surgery was superior to the prognostic value of the extent of disease (F values 27.00 and 12.69). Apparently, the preoperative C1-INH concentration is an essential and independent prognostic parameter of gastric carcinoma. We assume that C1-INH reflects an additional prognostic feature appropriate to the tumour or the host. Our finding that the interval between surgery and death from recurrence had an inverse relation to the preoperative C1-INH concentration also supports this assumption.

We have previously described the preoperative serum concentrations of immunoglobulins (Ig) and some complement components (C), and the erythrocyte sedimentation rate (ESR) in relation to the extent of disease and prognosis in patients with gastric carcinoma (Janssen *et al.*, 1983, 1985, 1987*a*). The levels of IgG decreased with advancing disease among those resected for cure, whereas C1-esterase inhibitor (C1-INH), C4 and ESR increased. Patients with recurrence had lower preoperative concentrations of IgG and higher concentrations of C1-INH than those alive and disease-free 2½ years after surgery.

The series of patients is now extended and the patients have been followed for 5 years, which permits the retrieval of further results. We have now searched for the exact predictive value of our variables as to recurrence or no-recurrence. The prognostic significance of the variables has also been compared with the prognostic significance of the extent of disease, which traditionally has been the prime indicator of prognosis (Nielsen *et al.*, 1985; Hartley *et al.*, 1987; Craven, 1987; Sobin *et al.*, 1988). Furthermore, we have also studied whether our variables were independent prognostic parameters or if they merely reflected the extent of disease at the time of surgery.

### Materials and methods

### *Patients*

The preoperative serum concentrations of IgG, IgA, IgM, C3, C4, C1-INH and the levels of ESR were quantified in 99 patients who underwent curative intent resection of gastric carcinoma in the Department of Surgery during the years 1977–82. Patients with other diseases or with a history of another malignant disease within the last 5 years before gastric cancer surgery were excluded. The mean age of the patients (± 1s.d.) was 66.1 ± 11.4 years and 40% were women.

After surgery the patients entered a regular follow-up programme with examinations every 3 months in the first year and later at 6-month intervals. The status of the series 5 years after surgery is shown in Table I.

During the first 5 years after surgery for gastric carcinoma, eight patients had a second primary cancer. Colon cancer appeared in three of them, urogential cancers in four and squamous cell lung cancer in one.

Ten patients died from various causes without signs of malignant disease at clincial or post mortem examination (three patients). One patient died from complications after a later laparotomy whereas nine patients died from cardiopulmonary or cerebrovascular diseases.

Three patients were lost on follow-up. They are all dead; at the last follow-up examination 1–3 months before death they were without signs of malignancy.

Five years after surgery 31 patients were alive and without clinical signs of disease. During follow-up 29 patients had a clinical course consistent with recurrence of gastric carcinoma. These two groups of patients were compared.

The median time between surgery and clinical signs of recurrence was 11.5 (range 2–59) months and the median time between recurrence and death was 4.0 (range 0–34) months.

The age difference between the patients with and without recurrence was small ($P>0.3$), and there was no sex difference between the groups ($P>0.2$). There was also no difference in the surgical procedures for total gastrectomy *vs* less extensive procedures ($P>0.1$) or splenectomy *vs* no splenectomy ($P>0.1$).

### *Pathology*

Based on the post-surgical criteria advised by IUCC (Harmer, 1978), the patients were divided into six groups of disease extent at the time of surgery: T1NOMO, T2NOMO, T3NOMO, T2N + MO, T3N + MO and T4NXMO (Nx, i.e. irrespective of lymph nodes). No patients with distant meta-

**Table I** Survey of 99 patients 5 years after potentially curative surgery for gastric carcinoma (1977–1982)

| | *No. of patients* |
|---|---|
| Alive and clinically cancer-free | 31 |
| Clinical course consistent with recurrance | 29 |
| Cancer in the vicinity of the resection border | 9 |
| Died in hospital after the operation | 9 |
| Died later from various causes without evidence of malignant disease | 10 |
| Second primary cancer after gastric cancer surgery | 8 |
| Lost on follow-up | 3 |

Correspondence: C.W. Janssen Jr, Department of Surgery, Haukeland University Hospital, N-5021 Bergen, Norway.
Received 3 January 1989; and in revised form 31 March 1989.

stases were resected for cure. There were no patients with T1N + MO disease in the series.

Tumours were also grouped into histological types according to Lauren's classification of intestinal type and diffuse (Lauren, 1965).

*Blood samples*

Concentrations of IgG, IgA and IgM and the complement components C3, C4 and C1-INH were quantified in sera as previously described (Janssen *et al.*, 1987*a*). ESR was routinely recorded on admission to the hospital.

*Statistics*

Mean values were compared by Student's *t* test, preceded by Fisher's test for comparison of variances. Distribution of nominal data was tested by $\chi^2$ test with Yates' modification. ESR was transferred to the ln-scale to fit better with a normal distribution.

The predictive value of the variables was tested in a discriminant analysis, performed by the program P7M in the BMDP statistical software (Dixon, 1983). The groups of disease extent were ranged in the order 1–6 and entered the discriminant analysis as an independent variable.

### Results

The patients with recurrence had lower preoperative concentrations of IgG and higher concentrations of C4 and C1-INH than the disease-free patients (Table II). These differences also held true within each group of disease extent. In each group of patients with and without recurrence, the concentrations of IgG, C4 and C1-INH had no significant variation with the extent of disease ($P>0.05$). ESR and the concentrations of IgA, IgM and C3 were not different between the patients with and without recurrence.

The disease extent was different between the patients with and without recurrence, with an excess of recurrences among the patients with lymph node metastases or T4 tumour, $0.005>P>0.001$. The distribution of Lauren's histological types of tumour was not different between the two groups of patients ($P>0.3$).

The potential of the variables to discriminate between the patients with and without recurrence was then tested. The extent of disease grouped in the order 1–6 entered the discriminant analysis. The most potent discriminator was C1-INH, with an F value more than twice as high as that of disease extent (Table III). The prognostic potential of IgG and C4 concentrations was slightly less than that of disease extent. Furthermore, the disease extent, IgG and C4 gave only insignificant additional information to that of C1-INH (F values were then respectively 3.46, 1.95, and 0.94).

With C1-INH as the only variable, 80% of the patients were classified correctly as to recurrence and non recurrence. The calculated critical C1-INH concentration was 0.378 g $l^{-1}$. Among 29 patients with C1-INH$\geqslant$ 0.38 g $l^{-1}$, 24 (83%) were in the recurrence group, whereas 24 patients out of 31 (77%)

**Table III** The potential (as F values) of disease extent (in groups 1–6) and the preoperative concentrations of IgG, C4 and C1-INH to discriminate between patients with and without recurrence after potentially curative surgery for gastric carcinoma

| *Variable* | *F value* | *Statistical significance (d.f. = 1,58)* |
|---|---|---|
| Disease extent | 12.69 | $P<0.001$ |
| IgG | 11.98 | $0.005>P>0.001$ |
| C4 | 7.10 | $0.01>P>0.005$ |
| C1-INH | 27.00 | $P<0.001$ |

Number of patients = 60.

with C1-INH$\leqslant$0.37 g $l^{-1}$ were alive and disease-free 5 years after surgery.

The C1-INH concentrations in the series ranged from 0.20 to 0.60 g $l^{-1}$. The calculated chance for recurrence at various levels of C1-INH is seen from Figure 1. With C1-INH at either the lower or upper end of the range, the chances for recurrence within 5 years after surgery were 2.6% and 98.9%, respectively.

The interval between surgery and death from recurrent gastric carcinoma decreased with increasing C1-INH levels preoperatively (Table IV). The median time between surgery and death from recurrence was 9 months when the preoperative C1-INH was >0.50 g $l^{-1}$ as opposed to 24 months for the patient with C1-INH <0.30 g $l^{-1}$.

Thirteen patients died from other causes without signs of malignant disease or were lost to follow-up. The mean concentrations of IgG, C4 and C1-INH in these patients were compared to those of the patients with and without recurrence (Gamel *et al.*, 1986). The values could not be assigned to any of the two groups.

**Figure 1** The calculated chance for recurrence of gastric carcinoma within 5 years after potentially curative surgery at different levels of preoperative C1-INH serum concentrations.

**Table II** Preoperative ESR and serum concentrations (mean ± 1s.d.) of immunoglobulins and complement components in 29 patients with recurrence and in 31 patients alive and disease-free 5 years after potentially curative surgery for gastric carcinoma.

| | *Cancer recurrence* | *No recurrence* | *Significance of differences* |
|---|---|---|---|
| lnESR(mm $h^{-1}$) | 2.73±0.77 | 2.74±0.87 | n.s. |
| IgG (g $l^{-1}$) | 8.50±2.37 | 11.43±3.94 | $0.002>P>0.001$ |
| IgA (g $l^{-1}$) | 2.29±1.18 | 2.25±1.19 | n.s. |
| IgM (g $l^{-1}$) | 1.22±0.60 | 1.23±0.58 | n.s. |
| C3 (g $l^{-1}$) | 0.92±0.27 | 0.86±0.16 | n.s. |
| C4 (g $l^{-1}$) | 0.46±0.13 | 0.37±0.11 | $0.01>P>0.005$ |
| C1-INH (g $l^{-1}$) | 0.42±0.07 | 0.33±0.06 | $P<0.001$ |

**Table IV** The median interval (months) between potentially curative surgery for gastric carcinoma and death from recurrence in 29 patients with various levels of preoperative C1-INH concentrations

| *C1-INH conc.* | *No. of patients* | *Time* |
|---|---|---|
| $\geq 0.20 \leq 0.30$ g $l^{-1}$ | 1 | 24 |
| $>0.30 \leq 0.40$ g $l^{-1}$ | 11 | 18 |
| $>0.40 \leq 0.50$ g $l^{-1}$ | 14 | 13 |
| $>0.50 \leq 0.60$ g $l^{-1}$ | 3 | 9 |

### Discussion

The preoperative serum concentrations of C1-INH, IgG and C4 were different between 29 patients with recurrence after potentially curative surgery for gastric carcinoma, and 31 patients who were alive and disease-free 5 years after surgery. The differences between the groups were consistent within each of six groups of disease extent. Furthermore, in each of the two groups of patients the variables were insignificantly associated with the disease extent. The between-group differences of our variables can therefore not be explained by the different stage distribution in the two groups.

When the prognostic significance of our variables was compared to that of the disease extent, we clearly showed that C1-INH was a more potent prognosticator than the disease extent. Actually, the disease extent gave no further prognostic information additional to C1-INH. We therefore assume that the preoperative serum concentration of C1-INH in patients with gastric carcinoma reflects an additional prognostic feature that is appropriate either to the tumour or the host.

It has been claimed that serum C1-INH parallels disease activity in cancer patients (Bach-Mortensen *et al.*,1975; Astrup *et al.*,1977; Koller *et al.*,1979). Our finding that among those with recurrence, the interval between surgery and death from recurrence was shorter where the preoperative C1-INH level was higher, also supports this opinion.

Serum IgG and C4 also gave insignificant prognostic information additional to C1-INH. This finding is in line with a previous report, where we described correlations between the concentrations of C1-INH and IgG, as well as C1-INH and C4 in gastric cancer patients (Janssen *et al.*, 1983). The preoperative ESR was nearly identical in the patients with and without recurrence. Any prognostic significance of ESR may have been confounded by the variations of ESR with the different histological types of tumour (Janssen *et al.*, 1987*b*), which was not the case. What we suppose is that ESR may reflect the tumour bearing state in one way or another.

Carcinoembryonic antigen (CEA) is a much used prognosticator of gastrointestinal malignancies. In patients with gastric carcinoma the preoperative CEA is clearly stage related (Janssen & Ørjasæter, 1986; Shimizu *et al.*, 1987; Koga *et al.*, 1987). The preoperative CEA concentrations were, however, only slightly different ($P < 0.05$) between patients with and without recurrence after gastric cancer surgery (Janssen & Ørjasæter, 1986), which may be ascribed to the variations of CEA with the histological types of tumour (Janssen *et al.*, 1987*b*).

We know of no other variable than the preoperative concentration of C1-INH that can predict so significantly the outcome after surgery for gastric carcinoma. This variable is easily obtained in a routine immunological laboratory at low cost and may be available before decisive therapy.

Two of us (Drs Janssen and Matre) dedicate this paper to our teacher and friend Professor Olav Tønder on the occasion of his retirement from his post as Professor of Immunology.

### References

ASTRUP, J., COLSTRUP, H. & FRANDSEN, B. (1977). Complement C1-inactivator in the serum of patients with malignant disease. *Acta Radiol. Ther. Phys. Biol.*, **16**, 394.

BACH-MORTENSEN, N., OSTHER, K. & STRØYER, I. (1975). C1-esterase inactivators and C4 in malignant diseases. *Lancet*, **i**, 499.

CRAVEN, J.L. (1987). Prognostic indices in stomach cancer. *Dev. Oncol.*, **48**, 322.

DIXON, W.J. (1983). *BMDP Statisical Software*. Revised edn. University of California Press: Berkely.

GAMEL, J., SEDDON, J., POLIVOGIANIS, L., ALBERT, D. & GREENBERG, R. (1986). A method for assessing potential bias among cancer patients recorded as 'Dead of other causes'. *Cancer*, **57**, 2246.

HARMER, M.H. (1978) *TNM Classification of Malignant Tumours*. 3rd edn. International Union against Cancer: Geneva.

HARTLEY, L.C., EVANS, E. & WINDSOR, C.J. (1987). Factors influencing prognosis in gastric cancer. *Aust. NZ J. Surg.*, **57**, 5.

JANSSEN, C.W. Jr, MAARTMANN-MOE, H. & LIE, R.T. (1987*a*). Preoperative prediction of extent and prognosis of gastric carcinoma by four serum proteins and erythrocyte sedimentation rate. *Eur. J. Surg. Oncol.*, **13**, 285.

JANSSEN, C.W. Jr, MAARTMANN-MOE, H. & LIE, R.T. (1987*b*). Concentrations of serum proteins and erythrocyte sedimentation rate in patients with different histological types of gastric carcinoma. *Eur. J. Surg. Oncol.*, **13**, 207.

JANSSEN, C.W. Jr & ØRJASÆTER, H. (1986). Carcinoembryonic antigen in patients with gastric carcinoma. *Eur. J. Surg. Oncol.*, **12**, 19.

JANSSEN, C.W. Jr, TØNDER, O. & MATRE, R. (1983). Stage-related correlations between immunoglobulins and complement components in preoperative sera from patients with gastric carcinoma. *Eur. J. Cancer Clin. Oncol.*, **19**, 1601.

JANSSEN, C.W. Jr, TØNDER, O. & MATRE, R. (1985). The prognostic value of preoperative serum immunoglobulin and complement component concentrations in patients with gastric carcinoma. *Acta Chir. Scand.*, **151**, 57.

KOGA, T., KANO, T., SOUDA, K., OKA, N. & INOKUCHI, K. (1987). The clinical usefulness of preoperative CEA determination in gastric cancer. *Jpn. J. Surg.*, **17**, 342.

KOLLER, M.E., HANEBERG, B., MATRE, R., FINNE, P.H. & ROMSLO, I. (1979). Lysozyme and complement factors in sera from children with acute lymphoblastic leukemia. *Acta Paediatr. Scand.*, **68**, 273.

LAUREN, P. (1965). The two histological main types of gastric carcinoma: diffuse and so-called intestinal-type carcinoma. *Acta Pathol. Microbiol. Scand.*, **64**, 31.

NIELSEN, J., AAGAARD, J. & TOFTGAARD, C. (1985). Gastric cancer with special reference to prognostic factors. *Acta Chir. Scand.*, **151**, 49.

SHIMIZU, N., WAKATSUKI, T., MURAKAMI, A. & 5 others (1987). Carcinoembryonic antigen in gastric cancer patients. *Oncology*, **44**, 240.

SOBIN, L.H., HERMANEK, P. & HUTTER, R.V.P. (1988). TNM Classification of malignant tumors. A comparison between the new (1987) and the old editions. *Cancer*, **61**, 2310.